# Tissue Engineering in Stomatology: A Review of Potential Approaches for Oral Disease Treatments

**DOI:** 10.3389/fbioe.2021.662418

**Published:** 2021-11-08

**Authors:** Lilan Cao, Huiying Su, Mengying Si, Jing Xu, Xin Chang, Jiajia Lv, Yuankun Zhai

**Affiliations:** ^1^ School of Stomatology, Henan University, Kaifeng, China; ^2^ Henan International Joint Laboratory for Nuclear Protein Regulation, Kaifeng, China

**Keywords:** tissue engineering, scaffolds, growth factors, periodontal, dental implants, cleft palate, oral and maxillofacial skin or mucosa, oral and maxillofacial bone

## Abstract

Tissue engineering is an emerging discipline that combines engineering and life sciences. It can construct functional biological structures *in vivo* or *in vitro* to replace native tissues or organs and minimize serious shortages of donor organs during tissue and organ reconstruction or transplantation. Organ transplantation has achieved success by using the tissue-engineered heart, liver, kidney, and other artificial organs, and the emergence of tissue-engineered bone also provides a new approach for the healing of human bone defects. In recent years, tissue engineering technology has gradually become an important technical method for dentistry research, and its application in stomatology-related research has also obtained impressive achievements. The purpose of this review is to summarize the research advances of tissue engineering and its application in stomatology. These aspects include tooth, periodontal, dental implant, cleft palate, oral and maxillofacial skin or mucosa, and oral and maxillofacial bone tissue engineering. In addition, this article also summarizes the commonly used cells, scaffolds, and growth factors in stomatology and discusses the limitations of tissue engineering in stomatology from the perspective of cells, scaffolds, and clinical applications.

## Introduction

In the 1980s, Professor Joseph P. Vacanti and Robert Langer from the United States first explored tissue engineering research (Vacanti et al., 1988). In 1993, they defined tissue engineering in an article as “an interdisciplinary field that applies the principles of engineering and the life sciences toward the development of biological substitutes that restore, maintain, or improve tissue function” (Langer and Vacanti, 1993).

**FIGURE 1:**
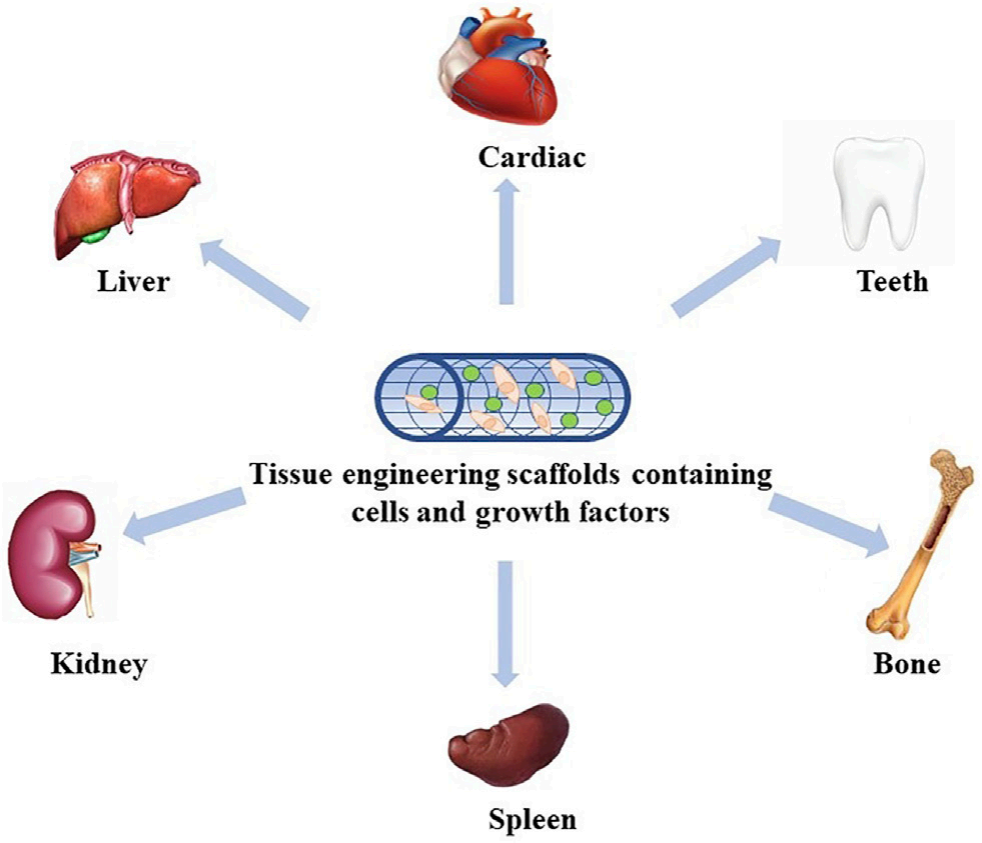
Application of tissue engineering. At present, tissue engineering has been widely used in many fields, including the heart, liver, kidney, spleen, bone, and teeth.

**FIGURE 2:**
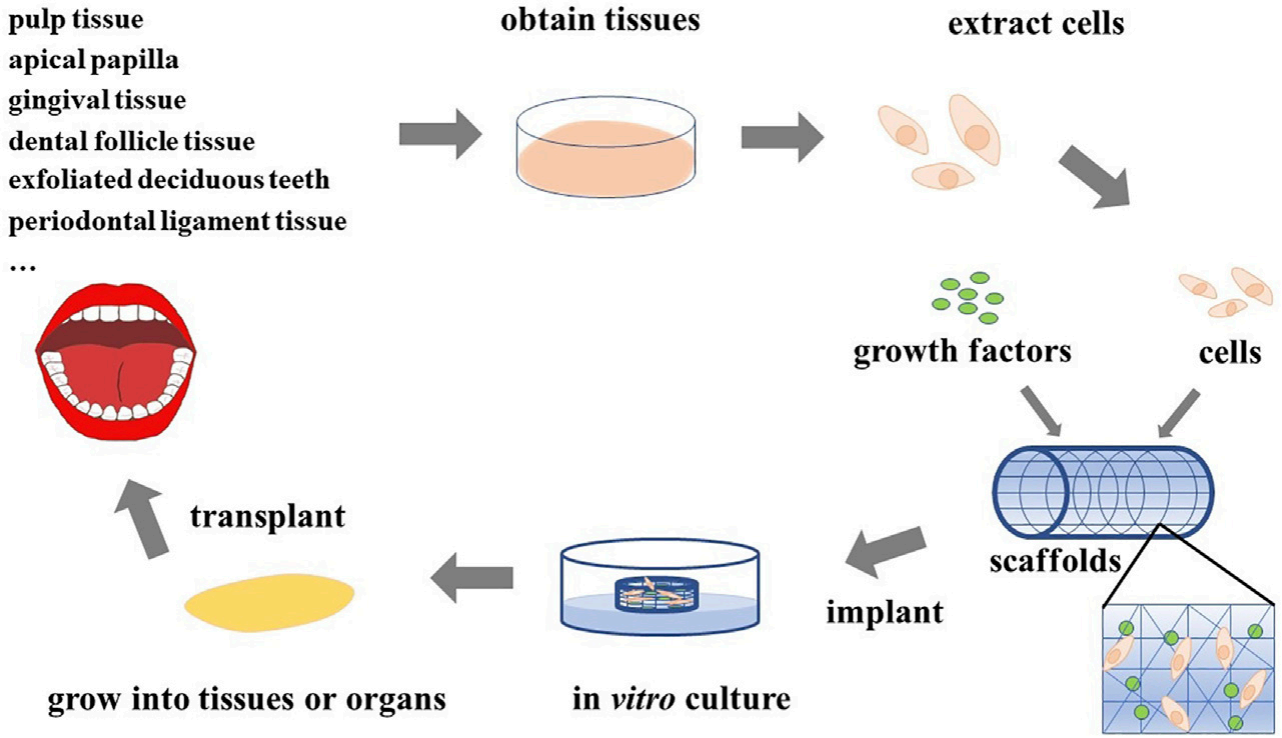
Principles of tissue engineering. Various cells extracted from the oral cavity are seeded on scaffolds adsorbed with growth factors, and the required tissues or organs can be obtained after appropriate *in vitro* culture and then implanted *in vivo*.

Nowadays, tissue engineering technology is booming and has become a popular research method for the reconstruction of damaged or missing tissues and organs (Fang et al., 2021; Farhat et al., 2021; Shang et al., 2021), and breakthroughs have been made in many fields (Figure 1) (Gosselin et al., 2018; Anandakrishnan and Azeloglu, 2020; Mirdamadi et al., 2020; Berbéri et al., 2021; Li et al., 2021; Scott et al., 2021). Therefore, we believe that tissue engineering technology will create extensive innovation in the field of stomatology. The basic principle of tissue engineering is to collect functionally related cells and plant them on a natural or synthetic scaffold with a certain spatial structure and induce cell proliferation through the influence of growth factors, thereby regenerating tissues or organs (Figure 2) (Han et al., 2014; Dzobo et al., 2018; Dey et al., 2020).

Cells are the source of biological activity in tissue engineering. Embryonic stem cells (ESCs) and adult mesenchymal stem cells (MSCs) are two types of stem cells classified according to their differentiation potential (Kolagar et al., 2020; Haghighat et al., 2021). Because of the ethical issues that limit the use of ESCs, multiple sources of MSCs have been more widely used in tissue engineering (Nancarrow-Lei et al., 2017). Induced pluripotent stem cells (iPSCs), which are obtained by artificially inducing somatic cells to express some specific genes, have the ability to divide indefinitely and hold a pluripotent differentiation capacity that enables them to differentiate into any human cells (Deicher and Seeger, 2021). In addition to bone mesenchymal stem cells (BMSCs) (Nakamura et al., 2013; Yoo et al., 2013; Selvasandran et al., 2018; Li Y. et al., 2019; Xu M. et al., 2019) and adipose-derived stromal cells (ADSCs) (Yoon et al., 2011; Yao et al., 2012; Mihaila et al., 2014; Zhu et al., 2019; Xu et al., 2020), various MSCs have also been derived from teeth in recent years (Volponi et al., 2010), such as dental pulp stem cells (DPSCs) (Chen Y.-Y. et al., 2016; Lambrichts et al., 2017), stem cells from human exfoliated deciduous teeth (SHEDs) (Alkaisi et al., 2013; Alipour et al., 2014; Behnia et al., 2014; Sugimura-Wakayama et al., 2015), periodontal ligament stem cells (PDLSCs) (Kim et al., 2010; Chen F.-M. et al., 2016; Panduwawala et al., 2017), stem cells from apical papilla (SCAPs) (Bakopoulou et al., 2011; Somoza et al., 2017; Yang et al., 2018; Yang et al., 2020; Shen et al., 2021), dental follicle cells (DFCs) (Tian et al., 2015; Yildirim et al., 2016; Lima et al., 2017), and gingival mesenchymal stem cells (GMSCs) (Zhang et al., 2009; Ansari et al., 2016; Shi et al., 2017; Rao et al., 2019; Liu X. et al., 2020) (Table 1). Scaffolds provide a suitable space for cell growth and functions. There are two main categories of scaffolding material used in tissue engineering research: natural and synthetic materials, such as ceramics, proteins, and polymers (Table 2) (Rai et al., 2015). Due to the limitations of single-type materials, composite scaffolds composed of two or more different materials have gradually attracted attention (Mogoşanu and Grumezescu, 2014). In recent years, the third-generation scaffolds are capable of promoting angiogenesis and inducing osteogenesis (Thein-Han and Xu, 2011). As carriers, scaffolds can provide sustained-release growth factors, which are soluble polypeptides that bind to cell membrane receptors (Pilipchuk et al., 2015). Some of these growth factors can promote epithelial regeneration, such as epidermal growth factor (EGF) (Zhao et al., 2010), and some induce bone formation such as bone morphogenetic protein (BMP), transforming growth factor-β (TGF-β), and basic fibroblast growth factor (bFGF). (Park et al., 2015), while others such as platelet-derived growth factor (PDGF) and vascular endothelial growth factor (VEGF) are beneficial in forming a functional vascular network (Table 3) (Yang et al., 2012). In conclusion, the core of tissue engineering lies in the establishment of a perfect three-dimensional spatial complex that consists of scaffolds, seed cells, and growth factors (Table 4).

**TABLE 1:** Cells commonly used in oral tissue engineering.

Cell	Sources	Functions	References
DPSCs	Pulp tissue	(1) Multidirectional differentiation potential; (2) play a paracrine effect on nerve cells and endothelial cells; (3) promote pulp regeneration; (4) expression of tendon markers under mechanical load	Chen et al. (2016b); Lambrichts et al. (2017)
SHED	Exfoliated deciduous teeth	(1) Extensive proliferation and differentiation ability; (2) enhance osteogenesis ability and repair bone defect; (3) inhibit the proliferation of T lymphocytes; (4) enhance peripheral nerve regeneration	Alkaisi et al. (2013); Alipour et al. (2014); Behnia et al. (2014); Sugimura-Wakayama et al. (2015)
PDLSCs	Periodontal ligament tissue	(1) Immunomodulatory effect on peripheral blood mononuclear cells of the same and heterogeneous species; (2) multidirectional differentiation potential and promotion of periodontal tissue regeneration; (3) treatment of periodontal bone defects	Kim et al. (2010); Chen et al. (2016a); Panduwawala et al. (2017)
SCAP	Apical papilla	(1) High proliferation rate and mineralization potential; (2) renewable dentin paste complex; (3) secrete TGF-β3; (4) capable of cloning and multiline differentiation; (5) express mesenchymal stem cell markers; (6) possesses the ability of cartilage differentiation and the potential to promote cartilage tissue regeneration	Bakopoulou et al. (2011); Somoza et al. (2017); Yang et al. (2018); Yang et al. (2020); Shen et al. (2021)
DFCs	Dental follicle tissue	(1) High proliferation potential; (2) excellent bone formation, fat formation and cartilage formation ability; (3) inhibit lymphocyte proliferation and apoptosis; (4) promote regeneration of dentin tissue; (5) express embryo, mesenchymal, and neural stem cell markers	Tian et al. (2015); Yildirim et al. (2016); Lima et al. (2017)
GMSCs	Gingival tissue	(1) Exhibit clonogenicity, self-renewal, and multipotent differentiation capacities; (2) immunomodulatory and anti-inflammatory component of the immune system *in vivo*; (3) promote tissue regeneration; (4) derived exosomes can promote wound healing and nerve regeneration; (5) regulate lipid metabolism and inflammation	Zhang et al. (2009); Ansari et al. (2016); Shi et al. (2017); Rao et al. (2019); Liu et al. (2020a)
ABMSCs	Alveolar bone	(1) Proliferation and differentiation ability; (2) improve the phagocytic activity of THP-1 macrophages; (3) inhibit the activation and proliferation of T lymphocytes; (4) excellent osteogenic differentiation ability and bone defect reconstruction ability	Liu et al. (2020b); Cao et al. (2020)
TGSCs	Tooth germ	(1) Affect the formation of new blood vessels, bone, fat and neurogenesis; (2) odontogenesis and osteogenesis	Yalvac et al. (2010); Taşlı et al. (2014); Ercal et al. (2017)
BMSCs	Bone marrow tissue	(1) The potential for self-renewal and multidirectional differentiation; (2) possesses fat-forming ability, bone-forming ability and angiogenesis ability; (3) promote wound healing; (4) secrete TGF-β and weaken the immune response in ischemic brain; (5) promote myocardial healing and improve heart function	Nakamura et al. (2013); Yoo et al. (2013); Selvasandran et al. (2018); Li et al. (2019b); Xu et al. (2019b)
ADSCs	Adipose tissue	(1) Good proliferation ability and cartilage differentiation potential; (2) promote fat formation; (3) osteogenic capacity; (4) paracrine function promotes blood vessel formation; (5) reduce the production of active oxygen and inflammation and improve skin photoaging	Yoon et al. (2011); Yao et al. (2012); Mihaila et al. (2014); Zhu et al. (2019); Xu et al. (2020)
NSCs	Primary tissues, somatic cells, and pluripotent stem cells	(1) Self-renewal and multidirectional differentiation ability; (2) potential to promote nerve regeneration	Bonaguidi et al. (2011); Ng et al. (2019)
ESCs	The early mammalian embryo	(1) Produce functional anterior pituitary gland; (2) excellent osteogenesis and angiogenesis ability; (3) rebuild epithelial tissue; (4) augment cardiomyocyte-driven heart regeneration	Ozone et al. (2016); Chen et al. (2018); Bargehr et al. (2019); Zhao et al. (2019)
iPSCs	SCAP, DPSCs, and SHED, gingival and periodontal ligament fibroblasts, and buccal mucosa fibroblasts	(1) Excellent osteogenesis and angiogenesis ability; (2) promote the formation of cementum, alveolar bone, and periodontal ligament to help PDL regeneration; (3) anti-inflammatory effects	Duan et al. (2011); Yang et al. (2014); Chen et al. (2018)

Abbreviations: DPSCs, dental pulp stem cells; SHED, stem cells from exfoliated deciduous teeth; PDLSCs, periodontal ligament stem cells; SCAP, stem cells from apical papilla; DFCs, dental follicle cells; GMSCs, gingival mesenchymal stem cells; ABMSCs, alveolar bone‐derived mesenchymal stem cells; TGSCs, tooth germ stem cells; BMSCs, bone marrow stromal stem cells; ADSCs, adipose‐derived stromal cells; NSCs, neural stem cells; ESCs, embryonic stem cells; iPSCs, induced pluripotent stem cells.

**TABLE 2:** Scaffolds commonly used in oral tissue engineering.

Type	Scaffolds	Advantages	Disadvantages	References
Naturally, derived polymeric scaffolds	Collagen	Favorable biocompatibility	Poor mechanical properties	Chattopadhyay and Raines (2014); Chang et al. (2016)
		Major protein of connective tissue	Unmanageable biodegradation rate	
		Low antigenicity		
	Alginate	Excellent biocompatibility	Not conducive to cell adhesion	Lambricht et al. (2014); Liao et al. (2017)
		Low cost	Low cell adhesion	
		Low immunogenicity		
	Chitosan	Favorable bioactivity	Slow degradation rate	Bhardwaj and Kundu (2012); Muzzarelli et al. (2015); Vishwanath et al. (2016)
		Low cytotoxicity	Inferior mechanical strength	
		Sterilizable; enhance bone and cartilage formation		
	Hyaluronic acid	Participate in various biological processes	Low mechanical strength	Lataillade et al. (2010); Ferroni et al. (2015); Chang et al. (2017)
		Turn over quickly	Complex structure	
		Bioactivity		
	Bioceramic	Excellent biocompatibility	Low biodegradability	Chang et al., (2017); Yu et al. (2017)
		Non-immunogenic	Inherent brittleness	
		Stable; high porosity		
Synthetic scaffolds	PEG	Favorable biocompatibility	Low cell reactivity	Zhu and Marchant (2011); Singh et al. (2013)
		Low cytotoxicity	Inert bioactivity	
		Great hydrophilicity	Non-biodegradability	
	PLLA	Great mechanical strength	Rapid degradation	Amjadian et al. (2016)
		Non-toxic biodegradable	Poor toughness	
	PLGA	Favorable biocompatibility	Inferior cell affinity	Gentile et al. (2014); Zhao et al. (2016b); Martins et al. (2018)
		Non-toxic biodegradable	Poor hydrophilicity	
		Allow to control the degradation rate	Swelling reaction of polymer	
	PCL	Excellent thermal stability	Inferior cell affinity	Siddiqui et al. (2018)
		Good mechanical properties	Poor hydrophilicity	
Composite scaffolds	Collagen and chitosan	Good flexibility	Chitosan is insoluble in water and most organic solvents	Fu et al. (2017); Lauritano et al. (2020); Wang et al. (2020)
		Reinforce the structure	Poor potentiality in cell adhesion/migration and proliferation	
		Increase pore size		
	HA-PLGA	Reduce the brittleness of the ceramics	Low degradation rates which cause exists longer time in cellular environment	Namini et al. (2018); Brassolatti et al. (2021)
		Better cell adhesion	Cellular responses are not sufficient.	
	PEG-PLGA	Accelerate periapical bone repair	Prohibitive cost	Shiehzadeh et al. (2014); Raddall et al. (2019)
		Biodegrade to carbon dioxide and water	Premix with autologous SCAP	

Abbreviations: PEG, polyethylene glycol; PLLA, poly(L-lactide) acid; PLGA, poly(lactic-coglycolic acid); PCL, polycaprolactone; HA, hydroxyapatite.

**TABLE 3:** Growth factors commonly used in oral tissue engineering.

Inducibility	Growth factors	Features	Oral applications	References
Pro-epithelialization	EGF	Induce stem cells to differentiate into epidermal cells	Promote the early healing of acute oral soft tissue wounds	Xing et al. (2013); Ben Amara et al. (2019)
		Promote the fibroblast proliferation		
Pro-osteanagenesis	BMP	Induce mineralization	Induce the differentiation of SHED into odontoblasts	Casagrande et al. (2010); Kim et al. (2012b); Agrawal and Sinha (2017)
		Bone and cartilage regeneration		
		Belong to TGF-β family		
	IGF	Initiate cell growth	IGF-1 family participate in the process of pulpal differentiation	Caviedes-Bucheli et al. (2009); Kim et al. (2012a); Magnucki et al. (2013)
		Induce cell proliferation		
		Combined with BMP2 can synergistically promote osteogenic differentiation		
	TGF-β	Regulate extracellular matrix synthesis	Stimulate odontoblast to secrete matrix	Wang et al. (2017); Weiss and Attisano (2013); Wang et al. (2017); Niwa et al. (2018)
		Induce fundamental cell processes such as proliferation, chemotaxis and apoptosis	Promote osteogenic differentiation of DPSCs	
Pro-angiogenesis	VEGF	The major factor for angiogenesis	Enhance proliferation and osteogenic differentiation of DPSCs *in vitro*	D' Alimonte et al. (2011); Shibuya (2013)
		Regulate endothelial cell secretion and proliferation		
	PDGF	Induce VSMCs proliferation and migration	A combination of collagen membrane and bone graft material mixed with rhPDGF-BB achieved alveolar ridge augmentation	Simion et al. (2012); Funato et al. (2013); Jin et al. (2014); Tan et al. (2015); Zhao et al. (2016a)
		Promote osteogenic differentiation	A collagen matrix infused with rhPDGF-BB increased the soft tissue volume in esthetic peri-implant sites	
		Induce MSCs chemotaxis and proliferation		
	FGF	Stimulate proliferation of fibroblasts and capillary endothelial cells	bFGF contributed to pulp cells proliferation and dentin matrix formation	Zhao et al. (2014); Baba et al. (2015)
		Promote angiogenesis and wound healing		
Pro-neurogenic	NGF	Regulate the growth and development of neurons	Induce the differentiation of immortalized dental papilla cells into odontoblasts *in vitro*	Arany et al. (2009)
		Facilitate axonal regrowth		

Abbreviations: EGF, epidermal growth factor; BMP, bone morphogenetic protein; IGF, insulin-like growth factor; TGF-β, transforming growth factor-β; VEGF, vascular endothelial growth factor; PDGF, platelet-derived growth factor; VSMCs, vascular smooth muscle cells; bFGF, basic fibroblast growth factor; NGF, nerve growth factor.

**TABLE 4:** Tissue engineering in stomatology.

Tissue engineering	Cells	Scaffolds	Growth factors	Applications	References
Tooth tissue engineering	DPSCs	PLLA	BMP	Obtain the mineralized crown	Cai et al. (2013); Iohara et al. (2013); Shiehzadeh et al. (2014); Baba et al. (2015); Tian et al. (2015); Wang et al. (2016a); Athirasala et al. (2018); Xu et al. (2019a); Nosrat et al. (2019); Oyanagi et al. (2019)
	IPSC	PLGA-PEG; alginate	FGF	Achieve pulp tissue regeneration	
	DFCs	Collagen-hydroxyapatite	IGF	Form biological root	
	PDLSCs;SCAP		CGF	Achieve functional whole-tooth restoration	
Periodontal tissue engineering	PDLSCs	PCL	CGF	Promote periodontal ligament, cementum, and alveolar bone regeneration; effectively repair periodontal defects	Dan et al. (2014); Fu et al. (2014); Zang et al. (2016); Duruel et al. (2017); Khodakaram-Tafti et al. (2017); Panduwawala et al. (2017); Yang et al. (2018); Aghamohamadi et al. (2020)
	SHED	PRF	IGF		
	SCAP	PLGA	BMP		
		HA/TCP; alginate; chitosan/ABB			
Dental implant tissue engineering	DPSCs;	HA	PRF	Change the alveolar bone and soft tissue environment; achieve good osseointegration and soft tissue augmentation	Wang et al. (2017); Simion et al. (2012); Funato et al. (2013); Hao et al. (2014); Yun et al. (2014); Wang et al. (2017); Iwasaki et al. (2019); Schorn et al. (2021)
	UCMSCs	Collagen	TGF-β		
	PDLSCs	Bioceramic	PDGF		
Cleft palate repair tissue engineering	iPSCs	PP	BMP	Closure of oronasal fistula; effectively guide palatal soft and hard tissue regeneration	Lipska et al. (2011); Kagami et al. (2014); Tarr et al. (2018); Von den Hoff et al. (2019); Schreurs et al. (2020); Tetè et al. (2020); Adhikari et al. (2021)
	CBSCs	PU; fibrin	CTGF		
	BMSCs	Alginate	EGF		
		Collagen	FGF		
		Polyesters; polyisocyanopeptide hydrogel	TGF-β		
Oral and maxillofacial skin or mucosal tissue engineering	ESCs; skin keratinocytes	PCL	EGF	Promote the epithelial regeneration of oral wounds; reconstruct oral skin and mucosa; improve aesthetics	Lubkowska et al. (2012); Peramo et al. (2012); Bayar et al. (2016); Nikoloudaki et al. (2020); Oliva et al. (2020); Toma et al. (2021)
	Oral mucosal epithelial cells	SPS	FGF		
		PLGA; collagen	PDGF		
		Tissue-engineered 3D cultures	VEGF		
Oral and maxillofacial bone tissue engineering	ADSCs;	Fibrin	HGF	Repair alveolar bone defect, maxillary bone defect, and mandibular defect; revascularization around maxillofacial bone	Khodakaram-Tafti et al. (2018); Redondo et al. (2018); Shahnaseri et al. (2020); Zhang et al. (2020); Liu et al. (2021)
	BMSCs;	BioMax	VEGF		
	ABMSCs	HA/TCP-β	SDF-1		
		Nanoporous HA	TGF-β1		

Abbreviations: ABMSCs, alveolar bone‐derived mesenchymal stem cells; ADSCs, adipose‐derived stromal cells; BMSCs, bone marrow stromal stem cells; CBSCs, cord blood stem cells; DFCs, dental follicle cells; DPSCs, dental pulp stem cells; ESCs, embryonic stem cells; iPSCs, induced pluripotent stem cells; PDLSCs, periodontal ligament stem cells; SHED, stem cell from exfoliated deciduous teeth; UCMSCs, human umbilical cord mesenchymal stem cells; PEG, polyethylene glycol; PLLA, poly(L-lactide) acid; PLGA, poly(lactic-coglycolic acid); PCL, polycaprolactone; HA, hydroxyapatite; PP, polypropylene; PU, polyurethanes; SPS, synthetic polymeric scaffolds; TCP, tricalcium phosphate; ABB, anorganic bovine bone; PRF, the patient-derived fibrin scaffold; EGF, epidermal growth factor; BMP, bone morphogenetic protein; IGF, insulin-like growth factor; TGF-β, transforming growth factor-β; VEGF, vascular endothelial growth factor; PDGF, platelet-derived growth factor; FGF, fibroblast growth factor; HGF, hepatocyte growth factor; SDF-1, stromal cell–derived factor.

## Tooth Tissue Engineering

The tooth, an indispensable organ to humans, consists of soft connective tissues, namely, the pulp in the pulp cavity, and three outer layers of mineralized hard tissue, such as enamel, cementum, and dentin, playing an important role in mastication, pronunciation, and aesthetics. Tooth development is accomplished by a series of epithelial–mesenchymal interactions and reciprocal inductions, which ultimately lead to cell differentiation and developmental space formation (Yuan and Chai, 2019). Tooth loss, which is caused by many reasons, such as dental caries, tooth agenesis, or trauma, is a common oral disease that seriously affects physiological functions and even increases the morbidity of gastrointestinal cancer (Ma et al., 2018), cardiovascular disease, and stroke (Cheng et al., 2018). Moreover, permanent teeth are not renewable once they fall off. At present, removable dentures and fixed dentures are commonly used in the clinic to repair missing teeth, but these traditional restorative methods suffer some flaws, such as causing discomfort and inefficient mastication (Hejazi et al., 2021). Hence, the construction of biological tissue-engineered teeth has emerged to solve these disadvantages. Tooth regeneration therapy for dental tissue repair and whole-tooth replacement has been a long-term goal to achieve in dentistry.

Researchers have already made some progress during the regeneration of partial dental tissues. Regenerative endodontics (RE) mostly utilize the strategy of cell homing and transplantation to repair or replace necrotic tissue and regenerate dentine–pulp complex (DPC) (Morotomi et al., 2019). First, the main principle of cell homing is that the body’s stem cells are recruited and induced to accumulate at the defective site, leading to endogenous tissue regeneration (Wang X. et al., 2018), but the mechanism and application prospects still require much research to clarify. In addition, cell transplantation is currently the main approach for achieving pulp tissue regeneration. A study combining pulp stem cells with granulocyte colony-stimulating factor (G-CSF) in a canine pulpectomy model found that pulp tissue containing vasculature and innervation filled the entire root canal, thereby achieving successful regeneration in pulp tissue (Iohara et al., 2013). There have also been some researchers attempting to develop a biomimetic tooth bud model with dental cells encapsulated within gelatin methacrylate (GelMA) hydrogel scaffolds to obtain a mineralized crown (Smith et al., 2017).

Simultaneously, whole-tooth bioengineering using embryonic tooth bud cells has been established in several animal models, including mice, rats, pigs, and dogs (Zhang and Chen, 2014). Cai et al. found that integration-free human urine–induced pluripotent stem cell (ifhU-iPSC)–derived epithelial sheets recombined with mouse dental mesenchyme could successfully regenerate tooth-like structures (Cai et al., 2013). Wang et al. proved the feasibility of whole-tooth regeneration in large animals by reconstructing single cells from the fourth deciduous molar tooth germ (p4) of pigs to bioengineer tooth buds in *in vitro* culture and *in vivo* transplantation in mouse subrenal capsules and jawbones. As a result, pig bioengineered tooth buds restore odontogenesis and develop into large tooth sizes (Wang F. et al., 2018). Ono et al. dissected canine permanent premolar (P2, P3, and P4) tooth germs from the mandible of beagles and then transplanted them into the alveolar bone socket of the same mandible to gain functional whole-tooth restoration by autologous transplantation of bioengineered tooth germ in a large animal model (Ono et al., 2017). Zhang et al. used decellularized tooth bud (dTB) scaffolds created from natural porcine tooth buds (TBs) and successfully formed mineralized whole teeth in miniature pig jaws *in vivo* (Zhang et al., 2017).

These results indicate that tissue-engineered teeth have bright prospects in tooth regeneration and can effectively solve the oral problems posed by tooth loss. In future, emerging technologies will provide increasingly advanced ideas for tooth regeneration.

## Periodontal Tissue Engineering

Periodontal tissue diseases are usually involved in periodontal inflammation and trauma, including destruction of the cementum, gingiva, periodontal ligament, and alveolar bone. The formation of periodontal pockets and the resorption of alveolar bone are typical manifestations of periodontitis and eventually develop into tooth loss. The most ideal periodontal treatment is to achieve complete functional regeneration of alveolar bone, cementum, and periodontal ligament to obtain new periodontal attachment (Iwata et al., 2014). Traditional periodontal therapy only removes bacteria and delays the disease process, but it is difficult to achieve periodontal regeneration. Different from traditional periodontal therapy, periodontal tissue engineering is a new concept for reconstructing defective periodontal tissues and organs and has already made rapid development in recent years.

The traditional tissue engineering methods are based on combining scaffolding materials with seed cells. Mrozik et al. cultured and purified sheep PDLSCs *in vitro*, combined them with gelatin sponges, and implanted them into the periodontal defect of the second premolar, and the newly formed alveolar bone, cementum, and Sharpey fibers were significantly more abundant than those in the control group without stem cell inoculation (Mrozik et al., 2013). Fu et al. treated animal models of periodontitis with stem cells isolated from miniature pig deciduous teeth (SPDs) plus hydroxyapatite/tricalcium phosphate (HA/TCP), and the loss of soft and hard tissue showed significant restoration after 12 weeks (Fu et al., 2014).

However, there are still differences between regenerated tissue and natural periodontal tissue in clinical applications (Matichescu et al., 2020). Therefore, newer techniques need to be introduced into the field of periodontal tissue engineering. Wu et al. inoculated gingival fibroblasts into Bio-Gide collagen membranes bilaterally and induced their mineralization, then constructed a tissue-engineered sandwich membrane to repair periodontal defects in premolar regions of beagles, and found that new alveolar bone, cementum, and periodontal ligament eventually formed (Wu et al., 2018). In terms of the processing and manufacturing of scaffolds, electrospinning technology is expected to provide more appropriate materials for tissue engineering. Higuchi et al. produced biodegradable membranes for the regeneration of periodontal tissue defects by electrospinning and sonocoating with nanohydroxyapatite particles (Higuchi et al., 2019). Sprio et al. fabricated hybrid superparamagnetic 3-layer scaffolds simulating the 3D environment of periodontium, which is conducive to boosting osteogenic and osteoconductive stimulation (Sprio et al., 2018). Regarding cell culture, cell sheet technology (CST) is defined as a cell transplantation method that does not require scaffolding materials and can preserve intact extracellular matrix (Sprio et al., 2018). Some researchers transplanted cell sheets supported by electrospun polycaprolactone (CaP-PCL) scaffolds, and denuded root and alveolar bone formation occurred at the defect site after 4 weeks, confirming that the combination of PCL and CaP-PCL scaffolds can promote periodontal regeneration (Dan et al., 2014). All these results provide important insights into advancements in periodontal tissue engineering, and it is believed that with the development of periodontal tissue engineering, complete realization of periodontal regeneration will be full of infinite possibilities.

## Dental Implant Tissue Engineering

**FIGURE 3:**
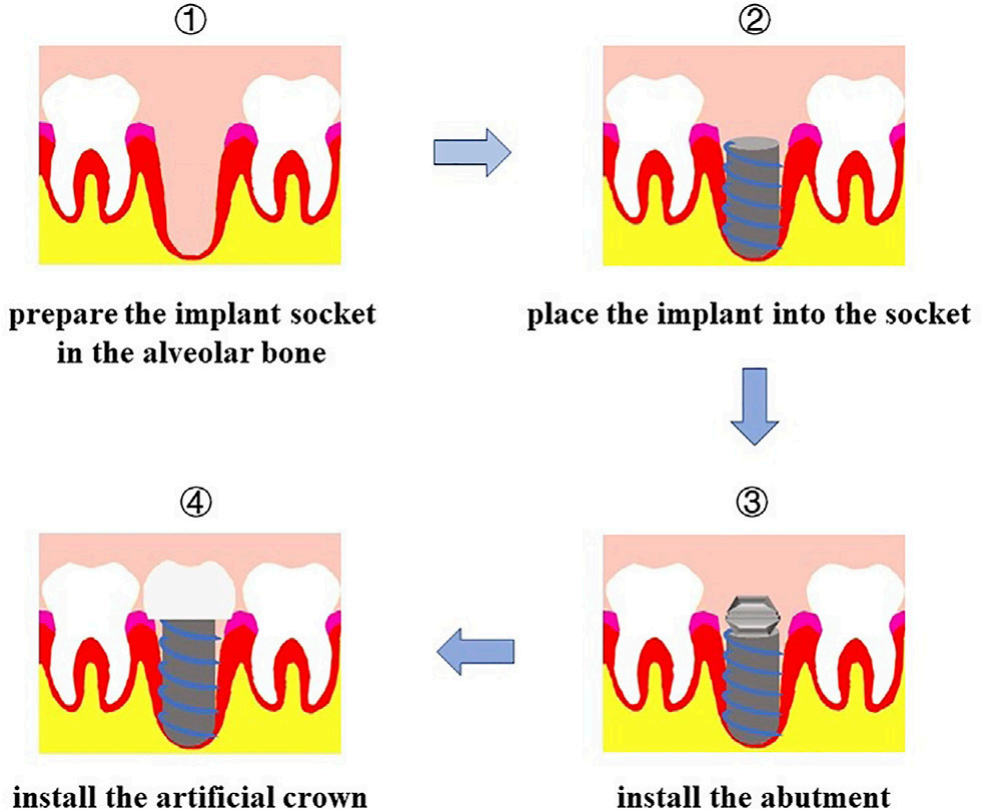
Procedure of dental implant. First, prepare the implant socket on alveolar bone; second, place the implant into socket; then install the abutment; finally, install the artificial crown.

We have mentioned the importance of teeth to humans and some related studies on the use of tooth tissue engineering to repair tooth loss. Dental implantation is another common method to restore tooth loss. Implant restoration is performed in the alveolar bone of the edentulous area to implant the artificial tooth root, which replaces the natural tooth root, and subsequently repair the absent the tooth, which includes the artificial crown of the upper part and lower part of the support of implants (Figure 3). Although dental implants overcome some disadvantages of dentures and effectively repair defects caused by tooth loss, two conditions still hinder the development of dental implant technology: 1) insufficient local bone mass in the implants (Pardal-Peláez et al., 2021) and 2) insufficient soft tissue around the implants (Noh et al., 2021). Dental implant tissue engineering mainly uses tissue engineering technology and changes the alveolar bone and soft tissue environment before the implant is implanted into the alveolar bone in the edentulous area to achieve good osseointegration (Hao et al., 2021) and soft tissue augmentation.

On the one hand, tissue engineering contributes to overcoming the obstacles encountered with bone regeneration during dental implants. Yun et al. applied platelet-rich plasma (PRP) and human bone marrow mesenchymal stem cells (BMMSCs) to the bone defect area around the dental implant with porous hydroxyapatite (HA) as the scaffold and determined the bone regeneration ability of BMMSCs and PRP histologically. The data showed that the HA + BMMSC + PRP group had a higher bone density between 6 and 12 weeks (Yun et al., 2014). To investigate the role of umbilical cord mesenchymal stem cells (UCMSCs) in bone defects around the implant after immediate implantation, Hao et al. filled the defect on one side with platelet-rich fibrin (PRF) and UCMSCs, while the other side was filled with PRF only as the control group and placed a titanium implant into the extraction socket. The results showed that UCMSCs can promote the formation of new bone in the bone defect area around implants; hence, UCMSCs can be used as excellent cells in the regeneration of bone defects after implantation (Hao et al., 2014).

On the other hand, concerning the problem of insufficient soft tissue, Simion et al. used a resorbable collagen matrix as a scaffold to carry recombinant human platelet–derived growth factor BB (rhPDGF-BB), and the results indicated that the soft tissue volume around implants increased moderately when applying a collagen matrix infused with rhPDGF-BB (Simion et al., 2012). Liu et al. employed acellular dermal matrix grafts conducive to increasing the attached gingiva and resin splint conducive to facilitating the healing of soft tissue attached to dental implants, and patients were satisfied with the reconstruction effects of dense connective tissue surrounding the implants after the operation (Liu et al., 2014). The patients with maxillary gingival recessions were treated with autologous fibroblast cell culture (AFCC) on a collagen scaffold placed under a coronally advanced flap (CAF), and soft tissues were significantly improved, suggesting that AFCC is a novel tissue engineering concept and a reliable therapy to solve the problem of insufficient soft tissues during defect repair caused by tooth loss (Milinkovic et al., 2015).

In summary, through the aid of tissue engineering technology, an increasing number of cells and scaffolds have been used for bone regeneration after dental implants, providing novel ideas for solving the problem of insufficient local bone mass in implants. Through the advantages of tissue engineering, such as less damage to the tissue around implants and good aesthetic effects, the development of oral implantology will be more vigorous in the future.

### Cleft Palate Repair Tissue Engineering

Cleft palate is one of the congenital malformations with the highest probability of occurrence in oral and maxillofacial regions and can occur alone or together with cleft lip. Cleft palate not only manifests as soft tissue deformity but also bone tissue defects and deformities and may be accompanied by disorders of jaw development. In other words, the occurrence of cleft palate will have a huge impact on facial esthetics, and it will also cause dysfunction in language, eating, and breathing. Therefore, the repair of cleft palate is crucial, and surgery is one of the most important therapy methods. Traditional palatoplasty usually applies a loose incision to reduce tension, but bone surface trauma exposed after surgery will be scarred and can even lead to the restriction of development and deformity of the jawbone (Cantarella and Mazzola, 2020; Choi et al., 2021).

To solve or avoid the problems caused by traditional surgical methods during the healing of cleft palate, researchers have tried to find better ways to resolve cleft palate. Tissue engineering technology has been applied to repair cleft palate and has already obtained some results in many studies. Bajestan et al. explored the use of *ex vivo* expanded stem cell populations to treat large alveolar bone defects in patients with a history of cleft palate or craniofacial trauma. The results indicated that stem cell population therapy is safe, but the ability to completely reconstruct large alveolar defects is finite, so further optimization is needed to satisfy the requirements of cleft palate treatment (Bajestan et al., 2017). Sharif et al. developed a plasma-functionalized electrospun composite polymer membrane, modified the fabricated membranes by plasma polymerization, and then implanted them in rats subcutaneously. The results showed that these membranes were biocompatible and angiogenic, providing the possibility for permanent closure of oronasal fistula (Sharif et al., 2019). Lee et al. created cell sheets derived from hMSCs and SHEDs for bone repair of cleft palate and found that the cell sheets led to calcification *in vitro*, which indicated that osteogenic stem cell sheets may become a new choice for the reconstruction of cleft palate (Lee J.-M. et al., 2019). Li et al. developed a tissue-engineered graft for the repair of cleft palate in young rats by incorporating and integrating a synthetic polymer with a human decellularized amniotic membrane (DAM). This cell-free and absorbable graft could effectively guide soft and hard tissue regeneration and support palate regeneration and tissue growth (Li W. et al., 2019).

In summary, the use of tissue engineering techniques to repair cleft palate not only avoids scar tissue formation, wound contraction, and facial deformity caused by traditional cleft palate repair surgery but also effectively reconstructs and stimulates the healing of defects. In other words, we believe that there may be a new breakthrough for the repair of cleft palate through the in-depth study of tissue engineering technology.

### Oral and Maxillofacial Skin or Mucosal Tissue Engineering

Skin and mucosal lesions caused by inflammation, trauma, tumors, or autoimmune diseases are very common in the clinical treatment of dentistry. Traditional autologous skin or mucosal flap transplantation is a popular method to treat lesions, but this method still has some disadvantages because the surgery causes donor site injury. Meanwhile, the source of homogenous skin or mucosal flap for transplantation is too limited, and the characteristics of exogenous tissue flap are different from oral and maxillofacial skin and mucosa. Even if the mucosal flap is successfully transplanted, it is difficult to maintain the secretion and lubrication function of the oral mucosa (Wang Z.-S. et al., 2016). To repair oral skin and mucosa lesions, an important task for researchers is to find alternatives to replace the traditional transplantation of autologous skin and mucosa, and the application of tissue engineering technology may provide a new direction in this research area.

Peramo et al. reported a three-dimensional tissue structure that can be used to repair lip defects, consisting of a continuous layer that contains the morphological features of lips: epidermal skin, vermilion, and oral mucosa, plus can produce tissues with similar anatomy as native human lips (Peramo et al., 2012). Yoshizawa et al. found that grafting *ex vivo*–produced oral mucosa equivalent (EVPOME) with live oral keratinocytes onto an intraoral mucosal wound can effectively promote epithelial regeneration in oral wounds (Yoshizawa et al., 2012). Bayar et al. created a construct containing a mucocutaneous junction with a transitional zone (vermilion) *in vitro*, which can produce a microvascular prelaminated flap in lip reconstruction, and the results showed that this construct could promote the phenotypic expression of regenerated tissue closer to native tissue (Bayar et al., 2016).

Some researchers preferred to combine flap surgery and tissue engineering technology to enhance the therapeutic effects in clinical treatment. Sieira et al. proposed a new approach to obtain keratinized mucosa over a fibula flap using full-thickness, tissue-engineered, autologous oral mucosa and found that this oral mucosa can restore native tissue and avoid peri-implant tissue complications during the repair of mucosal oral defects (Sieira Gil et al., 2015). Some research builds an oral mucosal model by using tissue engineering technology and evaluates the changes in the interface in implant soft tissue because the biotightness formed by the soft tissue around implants can impact the prognosis after dental implant treatments. Chai et al. developed a tissue-engineered three-dimensional oral mucosal model (3D OMM) by using primary human oral keratinocytes, fibroblasts, and a skin-derived scaffold. The titanium implant was then inserted into the engineered oral mucosa, and the results showed that the tissue-engineered oral mucosa was similar to the normal oral mucosa. 3D OMM can form epithelial attachments on the titanium surface (Chai et al., 2010). Trichloroacetic acid (TCA) has attracted the focus of dental researchers due to its pivotal role during skin regeneration. Lee et al. injected TCA into open wound defects of the palatal mucosa in beagles and found that TCA promoted the healing and regeneration of wound defects in oral soft tissue by upregulating cell cycle progression, cell growth, and cell viability (Lee K. et al., 2019).

The aforementioned studies demonstrated that tissue engineering technology can more easily repair defects in oral and maxillofacial skin or mucosa. If tissue-engineered skin and mucosa are widely used in oral and maxillofacial clinical surgery, it can effectively avoid the challenges caused by the transplantation of traditional autologous skin or mucosal flaps.

### Oral and Maxillofacial Bone Tissue Engineering

Oral and maxillofacial bone defects are diseases caused by congenital deformity, trauma, tumors, inflammation, or periodontal disease and mainly include alveolar, maxillary, and mandibular bone defects (Bangun et al., 2021; Lin and Kudva, 2021). Bone transplantation, guided bone regeneration membrane technology, stimulation of osteogenesis, and prosthetic repair are the main methods for the healing of defects. In the clinic, autologous bone is regarded as the “gold standard” for bone transplantation, but it also has some disadvantages. For example, autologous bone cannot be shaped randomly, which will impact the recovery and appearance of prognostic functions. Furthermore, the source is limited, and some complications may still occur after autologous bone transplantation. Recently, there have been many studies related to the healing of oral and maxillofacial bone defects by using bone tissue engineering technologies.

Khodakaram et al. compared the effects of fibrin glue scaffolds and autologous bone grafts during the healing of rabbit mandibular defects and found that they have similar osteogenic effects, so fibrin glue may be a good bone graft substitute and can be used to reconstruct maxillofacial bone defects (Khodakaram-Tafti et al., 2018). Shahnaseri et al. created a maxillary defect to simulate a human alveolar cleft model. One side of the defect was filled with hydroxyapatite/β-tricalcium phosphate scaffolds that contained mesenchymal stem cells from the subcutaneous adipose tissue of dogs, and the other side was filled with autologous bone grafts collected from the tibia. The results showed that both grafts had good bone formation effects, so tissue engineering can be used as an alternative method to reconstruct bone defects (Shahnaseri et al., 2020). Redondo et al. inoculated mesenchymal stem cells from alveolar bone into BioMax scaffolds prepared from autologous serum and treated maxillary cystic bone defects under GMP conditions. The results showed that BioMax cross-linked serum scaffolds containing osteogenic differentiated MSCs gained a good effect during the repair of maxillary defects (Redondo et al., 2018). Zhang et al. constructed tissue-engineered bones by using 3D printing molds and high-temperature sintering and produced nanoporous hydroxyapatite scaffolds that can convincingly repair *in situ* bone defects in experimental dogs (Zhang et al., 2020).

The reconstruction of bone defects (especially critical sized bone defects) is difficult because the survival and growth of bone require the surrounding and internal blood vessels to provide oxygen and nutrients. Therefore, the vascularization of tissue-engineered bone is very important during the repair of oral and maxillofacial bone defects. Matthias et al. successfully reconstructed large posttraumatic mandibular defects by using fresh frozen humeral allografts seeded with autologous bone marrow aspirate and vascularized them with a radial forearm flap (Matthias et al., 2019).

There are four main methods to reconstruct the blood supply of tissue-engineered bones: 1) using growth factors to promote the formation of new blood vessels (Omorphos et al., 2021); 2) culturing vascular endothelial cells as seed cells with the scaffold to form a complex unit and then implanting them *in vivo* to promote angiogenesis (Hancock et al., 2021); 3) combining microsurgery technology with bone tissue engineering to promote blood vessel formation (Vidal et al., 2020); and 4) using genetic engineering technology to promote blood vessel formation (Est-Witte et al., 2020). Selecting the appropriate tissue-engineered bone and constructing a good blood supply system will accelerate the healing of critical-sized bone defects. We believe that with the support of osteogenic cells, scaffolds, and growth factors, increasingly more tissue-engineered bone will be developed, and oral and maxillofacial bone defects will be repaired easily.

### Limitations

We mentioned that the basic elements of tissue engineering technology are cells, scaffolds, and growth factors. Current relevant studies also obtained satisfactory reconstruction results, but there are still some disadvantages that limit the development of tissue engineering. If researchers can understand these limitations of tissue engineering correctly, it will contribute to the further research and application of tissue engineering and will be helpful for solving problems during the healing of defective tissues or organs.

### Limitations of Cells

At present, the cells used for tissue engineering research mainly include xenogeneic cells, allogeneic cells, and autologous cells. Xenogeneic cells are taken from non-human body tissues and can be derived from animals such as pigs and dogs, which means that the use of xenogeneic cells may cause immune rejection. Although some researchers have overcome this immune rejection (Mohiuddin et al., 2014; Iwase et al., 2015), the safety and long-term therapeutic effects of xenogeneic cells still need to be further verified (Sun et al., 2019). Compared with xenogeneic cells, allogeneic cells can better overcome immune rejection (Goyer et al., 2019), but they may have some other disadvantages. In recent years, research on allogeneic cells has mainly focused on human embryonic stem cells derived from 1) naturally or artificially aborted embryos and 2) *in vitro* fertilized embryos. However, the application of human embryos is considered extremely cruel, immoral, and illegal in many countries. Autologous cells are taken from their own tissues and have the potential to regenerate various tissues and organs. Autologous cells, unlike xenogeneic and allogeneic cells, will not cause immune rejection and have no ethics problems, but their application is restricted by their limited source and traumas caused during cell harvesting.

### Limitations of Scaffolds

As previously summarized, natural biomaterials, synthetic polymer materials, or hydrogel scaffolds, all have some limitations. Because most natural biomaterials are derived from animal and have good biocompatibility during *in vivo* and *in vitro* experiments, they are still judged as non-autologous and labeled foreign bodies by the immune system and may eventually induce serious immunogenic responses after long-term use (Gilmartin et al., 2013). In addition, we should also pay attention to the instability of these biomaterials and the variability of molecular structures among different batches (Ige et al., 2012). Synthetic polymer materials generally exhibit poor cell affinity in previous studies (Zhao W. et al., 2016). The major disadvantage of electrospun scaffolds is the complexity of electrospinning and lack of defined control, so more reliable data from animal experiments are needed to support future practical applications (McClellan and Landis, 2016). Rasperini et al. reported the first human case in which a 3D-printed bioresorbable polymer scaffold was used to treat a periodontal osseous defect; however, the scaffold was exposed at 13 months and removed at 14 months because of a larger dehiscence and failure of wound healing (Rasperini et al., 2015). How to control the degradation rate of scaffolds to match the speed of defect healing and how to prepare layered scaffolds that can guide coordinated tissue regeneration may be the main directions of improvement approaches in the future.

### Limitations of Clinical Application

Constructing a tissue engineering complex rich in living cells *in vitro* and then implanting it *in vivo* is the main process of transplantation of engineered tissue or organs. However, it also has some potential risks to the recipients of implanted engineered tissues or organs. When culturing the engineered complex *in vitro*, it is necessary to add fetal bovine serum, streptomycin, or other substances that can promote cell growth, but most substances are not derived from humans themselves, so the engineered complex may cause allergic reactions after implantation *in vivo*. On the other hand, absorbable polymer materials and some other types of materials are often selected as scaffolding materials to support seeding cells. Although most of these materials show no toxic effects, the long-term safety and immunological rejection of these materials are still major concerns for clinical application. For example, people prefer using allogeneic bone as a scaffold material, but it still has little antigenicity even when treated at extremely low temperatures. Therefore, we should further consider the safety and validity of engineered tissue or organs before they are applied in the clinic.

## Conclusion

In summary, tissue engineering has broad prospects in stomatology and provides a valuable direction for future research on tooth loss, periodontal defects, dental implants, cleft palate defects, oral and maxillofacial skin or mucosal defects, and bone defects. It is believed that with the in-depth exploration of tissue engineering, ideal seed cell, better scaffold materials, and growth factors will be discovered and applied in effective clinical management of oral diseases in the future.
